# Development of Highly Efficient Lysosome‐Targeting Peptide Ligands for the Internalization of Extracellular Proteins

**DOI:** 10.1002/cbic.70587

**Published:** 2026-10-28

**Authors:** Yuan Zhao, Yaxian Liao, Xuankun Chen, Deqin Cai, Regina Stasser de Gonzalez, Hua Tang, Florence M. Brunel, Nick Cox, Joseph Stock, Guangsen Fu, Alexander N. Zaykov, Weiping Tang

**Affiliations:** ^1^ School of Pharmacy Lachman Institute of Pharmaceutical Development University of Wisconsin‐Madison Madison Wisconsin USA; ^2^ Department of Chemistry University of Wisconsin‐Madison Madison Wisconsin USA; ^3^ Chemical Biology Novo Nordisk US R&D Lexington Massachusetts USA

**Keywords:** IGF2R, LYTAC, protein internalization

## Abstract

Lysosome‐targeting chimeras (LYTACs) induce the internalization of extracellular and membrane‐associated proteins by routing them to lysosomes via endogenous receptors, such as the insulin‐like growth factor‐2 receptor (IGF2R), also known as cation‐independent mannose‐6‐phosphate receptor. While current IGF2R ligands are effective, expanding the LYTAC toolkit with structurally simple and synthetically accessible ligands remains a practical necessity. Here, we first validated that previously reported dimeric and monomeric peptides induce robust internalization, establishing them as effective IGF2R‐targeting LYTAC ligands. To further optimize the peptide architecture, we performed systematic mutational scanning on its monomeric sequence to simplify the peptide architecture. We identified the L4 variant, which features a single leucine substitution at the fourth position. This optimized monomer induces greater internalization efficiency than the previously reported wild‐type monomeric peptide ligand. To demonstrate its utility, we conjugated the L4 recruiter to a specific PCSK9 binder. The resulting bifunctional molecule successfully induced the dose‐ and time‐dependent internalization of PCSK9. Collectively, these findings establish several peptide variants as practical and highly effective building blocks for the development of extracellular protein internalization platforms.

## Graphical abstract

This study expands the targeted protein degradation toolkit by validating several structurally simple peptide recruiters. While wild‐type 431 monomeric and dimeric peptides serve as effective IGF2R‐specific modules, the optimized L4 variant emerges as a highly potent alternative that engages alternative endocytic pathways. By bridging extracellular targets to these respective receptors, these peptide‐based degraders drive robust endosomal internalization and subsequent lysosomal degradation.

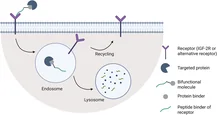

## 1 Introduction

Targeted protein degradation (TPD) is a promising therapeutic strategy that actively degrades disease‐causing proteins rather than merely inhibiting their function. While proteolysis‐targeting chimeras (PROTACs) effectively degrade intracellular targets via the ubiquitin‐proteasome system, they are generally limited to proteins with accessible intracellular domains [1, 4]. Because extracellular‐ and membrane‐associated proteins constitute a large portion of therapeutic targets, alternative approaches are needed [5]. To address this, lysosome‐targeting chimeras (LYTACs) were developed, which recruit endogenous endo‐lysosomal receptors to shuttle extracellular targets into lysosomes for degradation [6, 7, 8, 9, 10, 11, 12, 13, 14, 15, 16].

The insulin‐like growth factor‐2 receptor (IGF2R), also known as cation‐independent mannose‐6‐phosphate receptor (CI‐M6PR), is widely used for LYTAC‐mediated degradation. Current IGF2R‐recruiting ligands primarily include mannose‐6‐phosphate (M6P)‐based polymers, engineered IGF‐2 proteins, and IGF‐2 peptide mimics [6, 17, 18, 19, 20, 21, 22, 23]. Expanding the toolkit for IGF2R‐based LYTACs with additional synthetically accessible peptides would provide practical building blocks for the assembly of new degraders.

In this study, we started with a previously reported IGF2R‐binding peptide (431) [24]. We evaluated both the monomeric and dimeric versions of this peptide for their ability to induce the internalization of a large model cargo (NeutrAvidin‐650). Both peptides induced significant cargo internalization. Furthermore, we verified that the dimeric peptide effectively transports its cargo to the lysosome, confirming that these wild‐type peptides inherently serve as effective building blocks for IGF2R‐based LYTACs. To simplify the molecular structure while maintaining high delivery efficiency, we performed systematic single‐site mutational scanning of the monomeric sequence. This led to the identification of the L4 variant, peptide 10, where a single leucine substitution at the fourth position resulted in a markedly enhanced capacity to induce cargo internalization than the wild‐type monomer peptide 2. We then conjugated the L4 recruiter to a PCSK9 binder and confirmed that the resulting bifunctional molecule successfully induced the dose‐ and time‐dependent internalization of PCSK9. This structurally simple L4 variant emerges as a highly efficient and robust peptide platform for future TPD.

## 2 Results and Discussion

### 2.1 Design and Screening of IGF2R‐Recruiting Peptides for Bifunctional Molecule Development

To identify an efficient IGF2R‐recruiting ligand, we started with the previously reported IGF2R‐binding peptide, 431 [24]. We synthesized a panel of five biotinylated variants (peptides 1–3, Figure 1A) to test their ability to drive the cellular uptake of a model cargo, NeutrAvidin (NA‐650) (Figure 1B).

**FIGURE 1:**
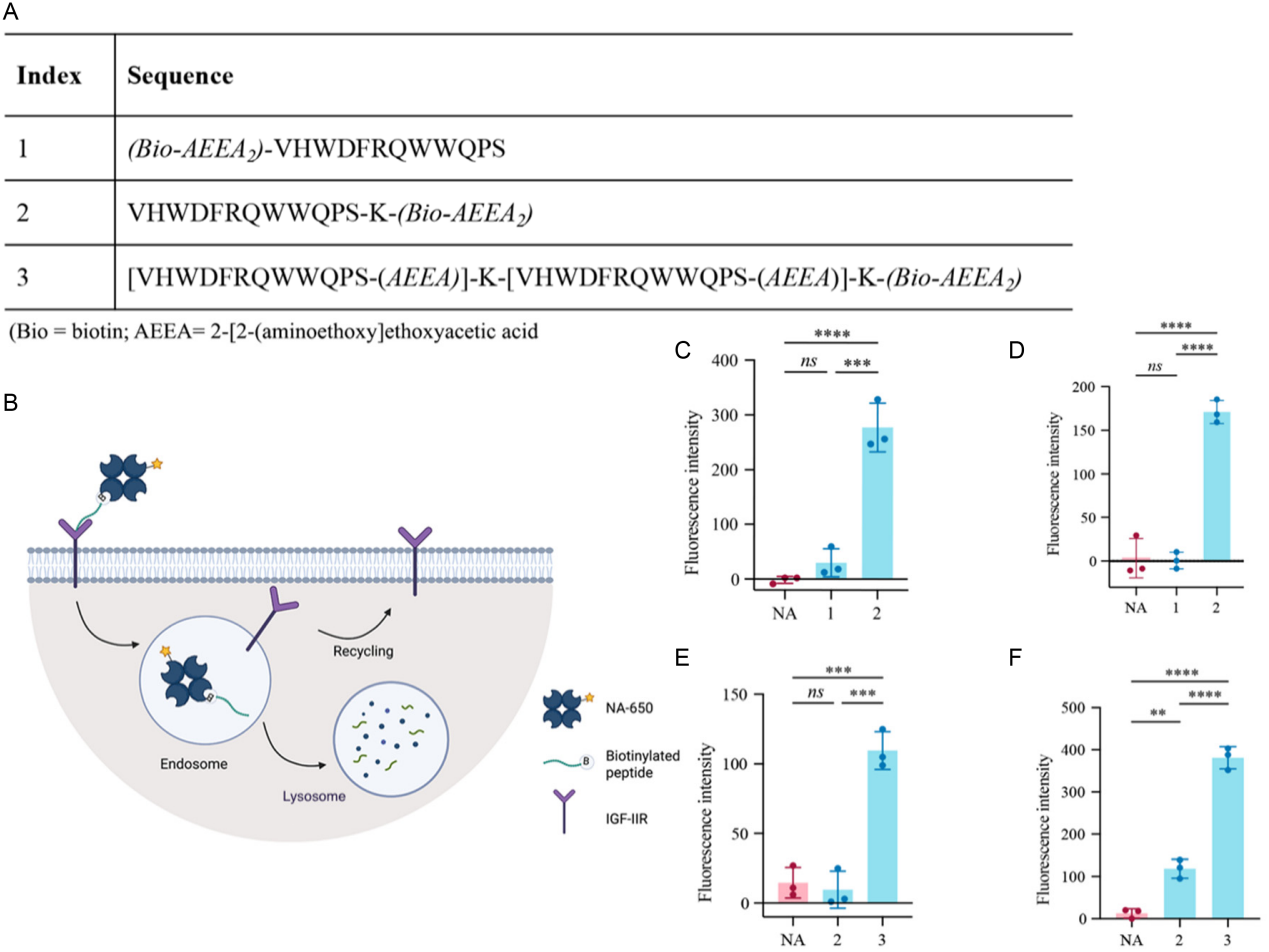
Design and evaluation of monomeric and dimeric IGF2R‐recruiting peptides for targeted cargo internalization. (A) Sequences and structural compositions of the synthesized biotinylated peptides (peptides 1–3). (B) Illustration of targeted cargo (NA‐650) internalization induced by biotinylated peptides. (C,D) Comparison of cellular uptake of the model cargo (NA‐650, 125 nM) driven by peptides 1 (500 nM) and 2 (500 nM) in HepG2 (C) and Huh7 (D) cell lines. Data are presented as mean ± SD. The statistical significance was assessed using ordinary one‐way ANOVA test, ***p < 0.001, ****p < 0.0001, ns: not significant (*n* = 3 biological independent samples). (E,F) Comparison of recruitment efficiency between monomeric peptide 2 (500 nM) and the dimeric peptide 3 (500 nM) at varied cargo concentrations (25 nM (E) and 125 nM (F)) in HepG2 cells. Data are presented as mean ± SD. The statistical significance was assessed using ordinary one‐way ANOVA test, **p < 0.01, ***p < 0.001, ****p < 0.0001, and ns: not significant (*n* = 3 biological independent samples).

First, we examined how the biotin attachment site affected monomer activity. Comparing N‐terminal (peptide 1) and C‐terminal (peptide 2) biotinylation via an *AEEA* linker, we found that peptide 2 induced significantly higher internalization (Figures 1C,D and S1A). This indicated that C‐terminal modification better preserves the function of the 431 motifs.

Previous studies have demonstrated that dimerization of the 431 peptide significantly enhances its intrinsic cellular uptake. To determine whether this multivalent strategy also translates to improved cargo delivery, we synthesized biotinylated dimeric 431 peptide (peptide 3) connected by *AEEA* spacers. Consistent with prior reports on the peptide alone, the dimeric peptide 3 drove significantly higher cargo uptake than the monomeric peptide 2 (Figures 1E,F and S1B,C). Based on these results, we selected peptide 3 as our dimeric benchmark for subsequent studies.

### 2.2 Structural Evaluation of the Dimeric Recruiter

Following the identification of the 431‐dimer (peptide 3) as our lead scaffold, we performed a systematic structure–activity relationship (SAR) study to evaluate the impact of specific residues, linker density, and overall molecular architecture (peptides 4–9, Figure 2).

**FIGURE 2:**
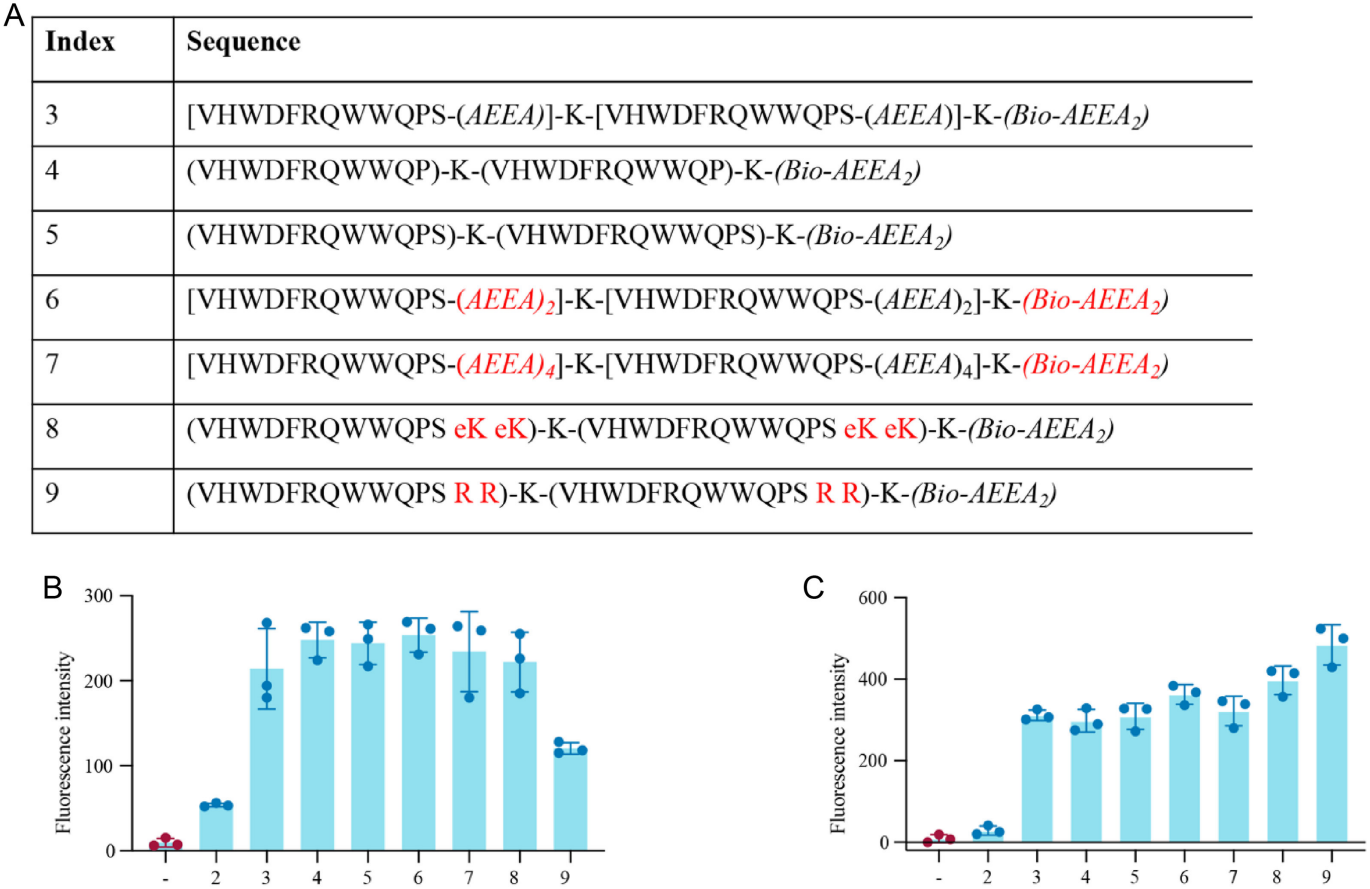
Structure‐activity relationship (SAR) analysis of dimeric IGF2R recruiters. (A) Sequences and structural modifications of the synthesized biotinylated dimeric peptides (peptides 6–11), exploring C‐terminal residues, linker length, and surface charge. (B,C) Fluorescence quantification using a microplate reader comparing cellular uptake of model cargo (NA‐650, 25 nM) driven by dimeric variants 4–9 in Huh7 cells at two concentrations: 100 nM (B) and 500 nM (C). peptide 2 (WT monomer) and peptide 3 (WT dimer) serve as benchmarking controls. Data are presented as the mean ± SD (*n* = 3 biological independent samples).

We first examined the role of the C‐terminal serine (S) and observed that its removal (peptide 4) did not significantly impair uptake, suggesting flexibility at the peptide’s distal end. We further investigated the impact of spatial separation between the two 431 motifs by modulating the number of *AEEA* units. When comparing a linker‐free construct (peptide 5) with variants containing one, two, and four *AEEA* units per monomer (peptides 3, 6, and 7), we observed no significant differences in cargo internalization efficiency. This indicates that the dimeric scaffold can accommodate moderate variations in linker length without compromising its endocytic activity. Furthermore, we explored the influence of surface charge by incorporating lysine–lysine (peptide 8) or arginine–arginine (peptide 9) motifs. At a concentration of 500 nM, the introduction of arginine residues in peptide 9 resulted in only a marginal increase in fluorescence intensity. Furthermore, to minimize potential nonspecific membrane interactions associated with excessive positive charge, this modification was not pursued. Collectively, these findings reaffirmed peptide 3 as a highly robust and functional dimeric baseline for IGF2R recruitment.

### 2.3 Characterization of 431‐Dimer‐Mediated Cargo Internalization and Lysosomal Trafficking

With peptide 3 selected as our dimeric recruiter, we next characterized its ability to drive the cellular uptake of the model cargo, NA‐650. Time‐course experiments demonstrated a time‐dependent accumulation of intracellular NA‐650 over 24 h (Figure 3A). Western Blot analysis also showed a dose‐dependent increase in internalized NA‐650, reaching a plateau around 0.63 μM (Figure 3B). Importantly, evaluating total IGF2R levels via Western Blot revealed no receptor degradation. This indicates that peptide 3 successfully drives cargo internalization without depleting the endogenous receptor. To assess extracellular clearance, we quantified the remaining NA‐650 in the culture media. Treatment with 1000 nM peptide 3 depleted approximately 80% of the cargo from the media compared to control conditions (Figure 3C).

**FIGURE 3:**
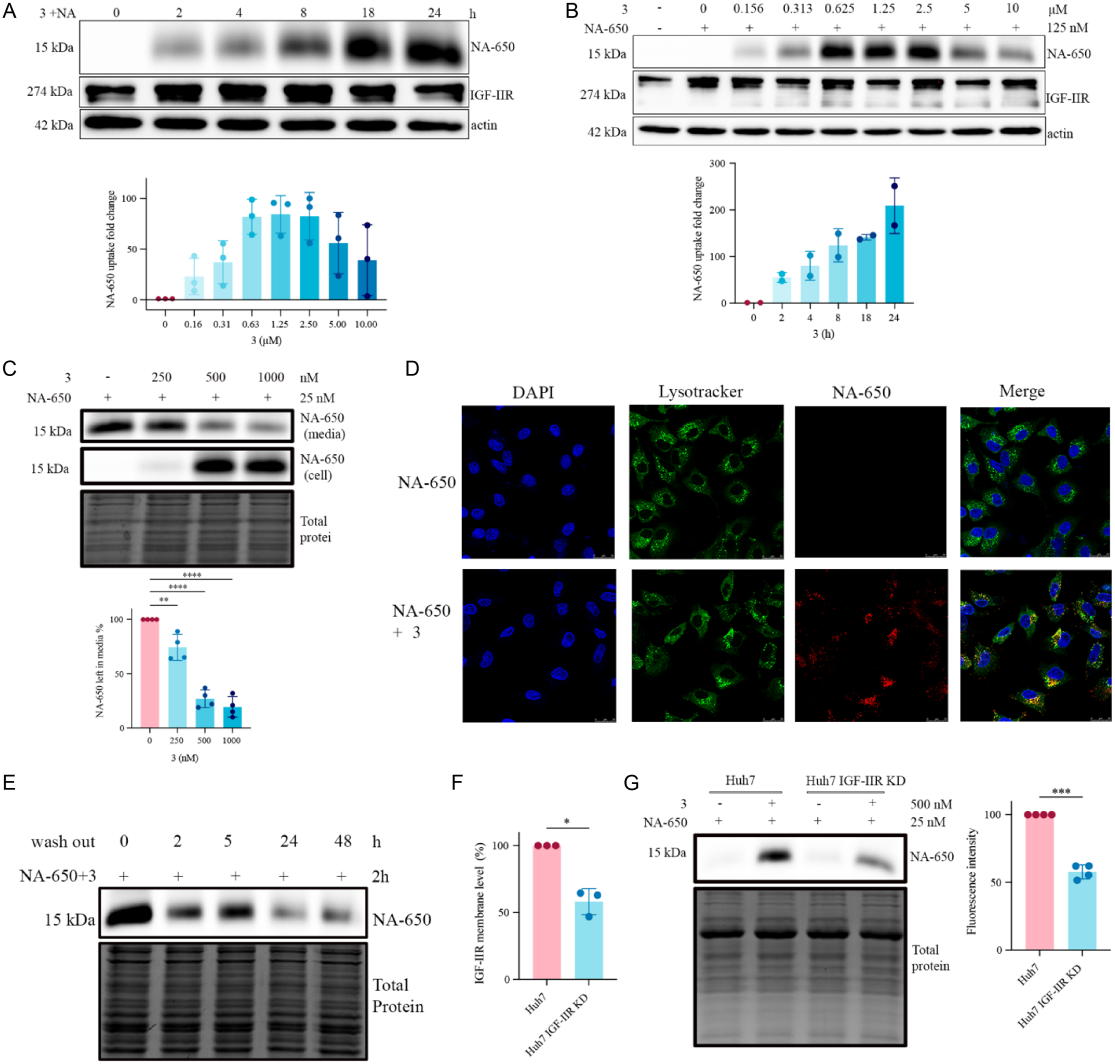
Characterization of peptide 3‐mediated cargo internalization. (A) Time‐course of NA‐650 (125 nM) cellular uptake (0–24 h) driven by peptide (500 nM) in HepG2 cells. Data are presented as the mean ± SD (*n* = 3 biological independent samples). (B) Concentration‐dependent internalization of NA‐650 in HepG2 cells treated with varied concentrations of peptide 3 (0–10 μM) and fixed NA‐650 (125 nM). Data are presented as the mean ± SD (*n* = 2 biological independent samples). (C) Depletion of NA‐650 from the culture media and corresponding intracellular accumulation upon peptide 3 treatment in Huh7 cells. Data are presented as mean ± SD. The statistical significance was assessed using ordinary one‐way ANOVA test, **p < 0.01, ***p < 0.001, and ****p < 0.0001 (*n* = 3 biological independent samples). (D) Confocal microscopy analysis of NA‐650 (25 nM) intracellular uptake and lysosomal colocalization mediated by peptide 3 (500 nM) in Huh7 cells (blue, DAPI; green, lysotracker; and red, NA‐650). (*n* = 3 biological independent samples). (E) Postinternalization clearance of NA‐650 following washout after 2 h treatment with the L4‐based bifunctional molecule. (*n* = 3 biological independent samples). (F) Quantification of cell‐surface IGF2R levels to verify receptor knockdown (KD) in the Huh7 cell line. Data are presented as mean ± SD. The statistical significance was assessed using an unpaired two‐tailed *t* test, *p < 0.05 (*n* = 3 biological independent samples). (G) Western blot analysis and corresponding fluorescence quantification of internalized NA‐650 in wild‐type (WT) versus IGF2R KD Huh7 cells treated with peptide 3 and NA‐650, demonstrating the receptor‐dependent uptake of the cargo. Data are presented as mean ± SD. The statistical significance was assessed using ordinary one‐way ANOVA test and an unpaired two‐tailed *t* test,***p < 0.001 (*n* = 4 biological independent samples).

Next, we tracked the intracellular localization of the internalized NA‐650 using confocal microscopy to confirm its delivery to lysosomes. While NA‐650 alone showed negligible cellular entry, adding peptide 3 induced significant cargo internalization. Costaining with LysoTracker revealed colocalization between the internalized NA‐650 and lysosomes, appearing as yellow puncta in the merged images (Figure 3D). This confirms that peptide 3 successfully routes its cargo to lysosome. To determine the postinternalization fate of the recruited cargo, we performed a washout experiment following a 2 h treatment with the L4‐based bifunctional molecule (Figure 3E). After removal of the extracellular compound, cells were cultured in compound‐free medium for up to 48 h, and the intracellular cargo level was monitored over time. A progressive reduction in the intracellular NA‐650 signal was observed following washout, with substantially lower levels detected at 24 and 48 h compared with the initial posttreatment time point, while total protein levels remained comparable across samples. These results indicate that the internalized cargo does not simply accumulate intracellularly but is progressively cleared following uptake, supporting postinternalization clearance of the recruited protein.

Together with the efficient cargo internalization mediated by the optimized L4 ligand, these findings further support the potential of this peptide scaffold for the development of functional lysosome‐targeting bifunctional molecules.

Finally, to verify if this uptake depends on IGF2R, we generated a stable IGF2R knockdown (KD) Huh7 cell line using an sgRNA‐mediated approach. Flow cytometric analysis confirmed an approximately 40% reduction in cell‐surface IGF2R expression compared to wild‐type controls (Figure 3F). When we compared NA‐650 internalization between the wild‐type and KD cells, we observed a corresponding ~50% decrease in fluorescence signal in the receptor‐deficient cells (Figure 3G). This partial inhibition of cargo uptake aligns with the reduced levels of available surface receptors. Together, these data confirm that peptide 3‐mediated cargo delivery is indeed dependent on the IGF2R pathway, consistent with previously reported binding studies [24].

### 2.4 Comparison of Peptide 3 With Other Established IGF2R Ligands

To evaluate peptide 3 relative to existing tools, we compared its internalization efficiency with two established IGF2R‐targeting ligands: a hexametric mannose‐6‐phosphonate (M6Pn) conjugate and the wild‐type IGF‐2 protein [20, 22].

Western blot analysis in HepG2 and Huh7 cells showed that peptide 3 induced much more NA‐650 internalization than the M6Pn‐based recruiter (Figure 4A,B). Specifically, peptide 3 at 1 μM resulted in a stronger intracellular signal than M6Pn‐biotin at 10 μM. Furthermore, when tested at the same concentration, peptide 3 induced higher cargo uptake than the wild‐type IGF‐2 protein across both cell lines (Figure 4C,D). Together, these results confirm that peptide 3 is a highly effective IGF2R recruiter compared to current synthetic and endogenous standards, supporting its use for the subsequent design of bifunctional molecules.

**FIGURE 4:**
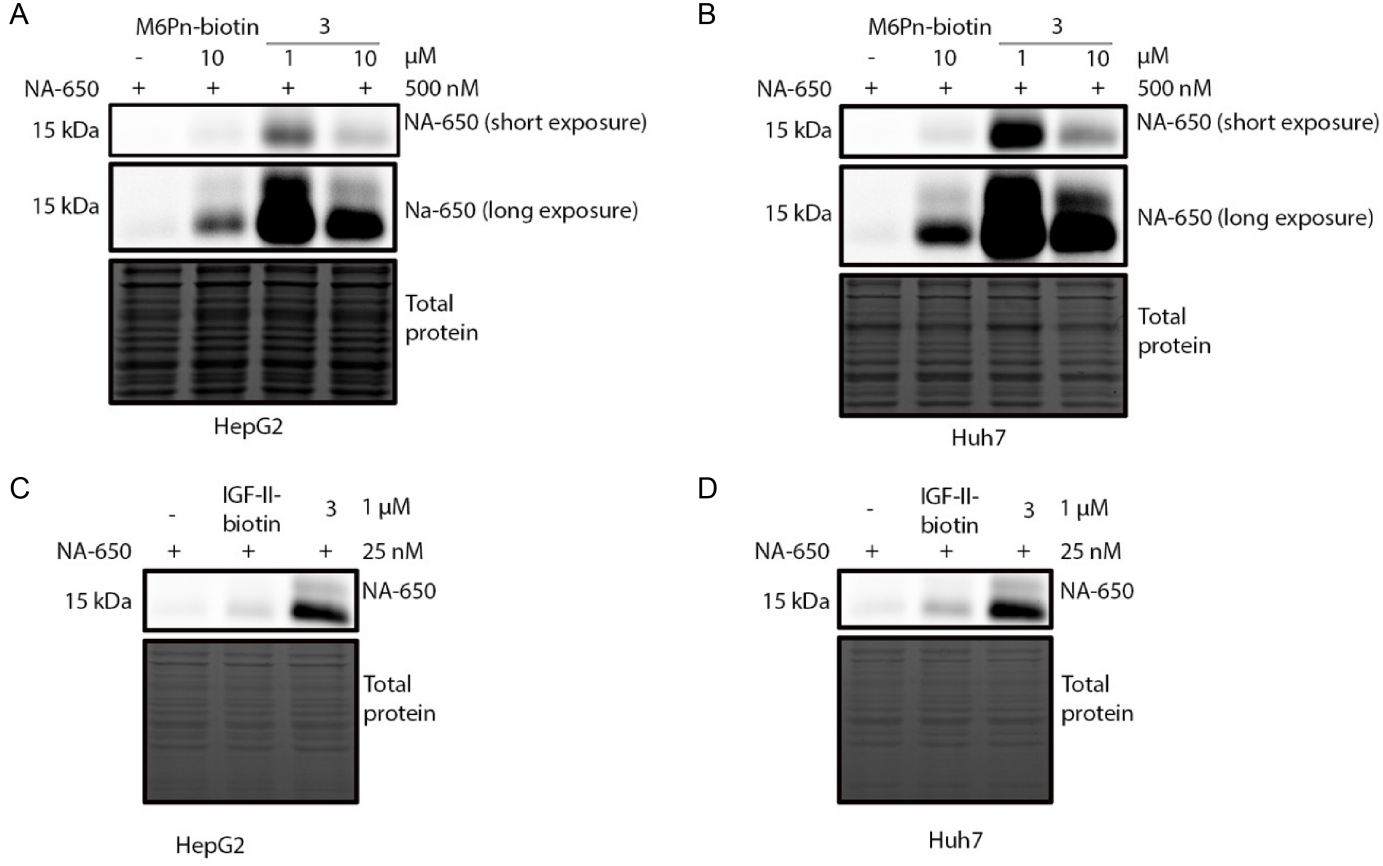
Comparative uptake efficiency of peptide 3 versus canonical IGF2R ligands. (A,B) Comparative internalization of NA‐650 mediated by peptide 3 versus M6Pn‐biotin in HepG2 (A) and Huh7 (B) cells. (*n* = 3 biological independent samples). (C,D) Comparative internalization of NA‐650 mediated by peptide 3 versus IGF2‐biotin in HepG2 (C) and Huh7 (D) cells. (*n* = 3 biological independent samples).

### 2.5 Mutational Scanning of the Monomeric 431 Peptide

To determine whether we could improve the recruitment efficiency of the 431 monomer, we performed a mutational scan. We synthesized a library of variants based on the 12‐amino‐acid 431 sequence. For positions 2 through 12, each residue was individually substituted with eight representative amino acids. At the first position, the library included four wild‐type replicates as internal controls alongside four specific substitutions (Figure 5). The impact of these point mutations was evaluated by measuring NA‐650 uptake in both Huh7 and U87 cells. The resulting heatmaps showed consistent activity patterns across both cell lines, highlighting residues that are critical for IGF2R engagement.

**FIGURE 5:**
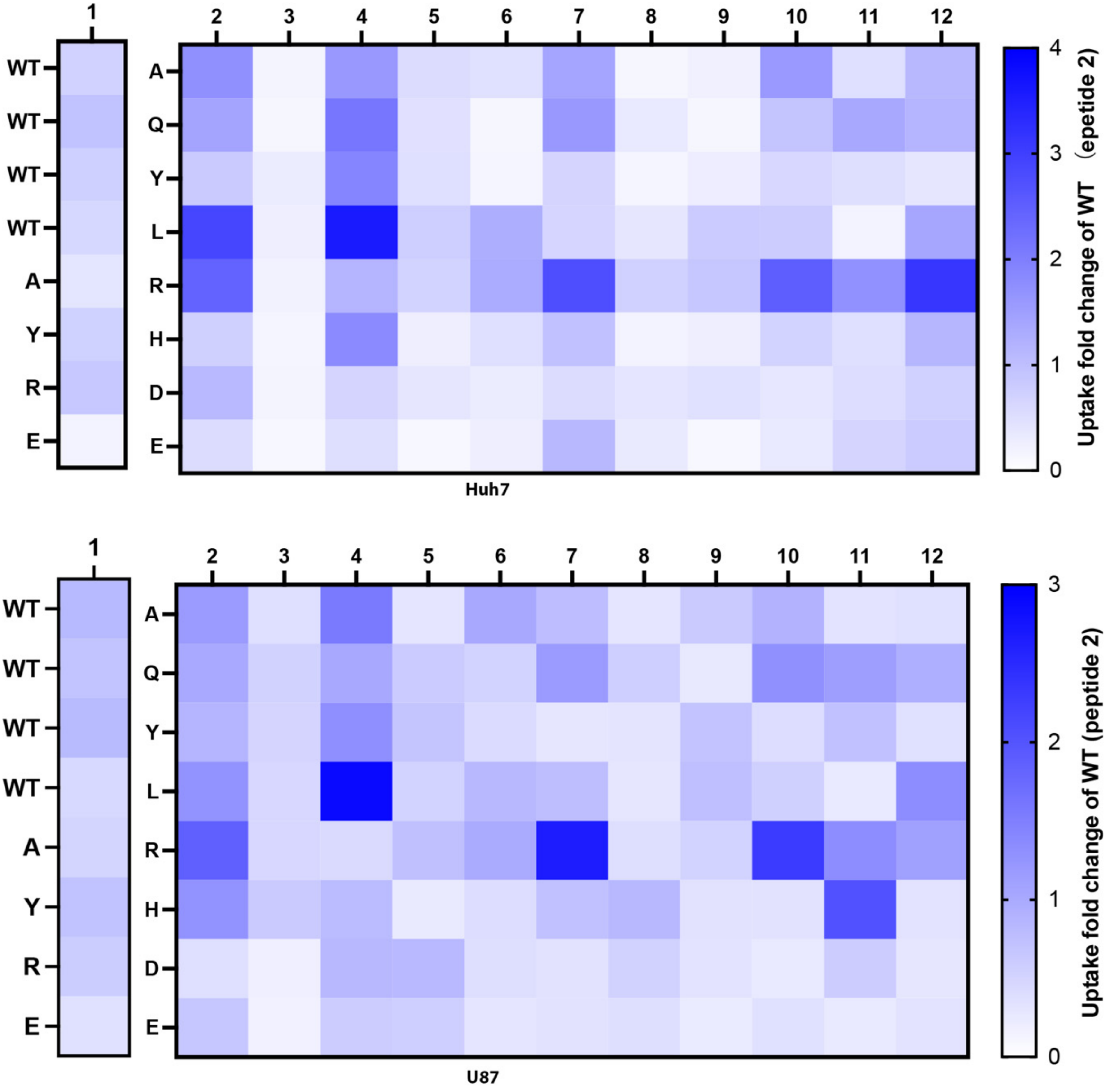
Mutational landscape and optimization of the IGF2R recruiter peptide. (A,B) Heatmap analysis of relative NA‐650 uptake (fold change over wild‐type peptide 2) mediated by single‐amino‐acid substituted peptide variants in Huh7 (A) and U87 (B) cell lines. The horizontal axis represents the specific residue positions (1–12) being mutated, and the vertical axis indicates the new amino acids introduced at these positions. Darker blue indicates enhanced internalization efficiency compared with the wild‐type (WT) baseline.

While most substitutions either maintained or reduced internalization, a specific mutation at the fourth position significantly enhanced cargo uptake. Substituting the wild‐type residue with leucine at position 4 (the L4 mutation) resulted in the highest internalization efficiency in both Huh7 and U87 cells (Figure 5). A few other positions, such as positions 7 and 10, showed slight improvements when substituted with basic amino acids like arginine (R), but the L4 substitution remained the most potent single modification.

Based on these screening results, we selected this optimized L4 monomer as our lead sequence for subsequent bifunctional molecule design.

### 2.6 Comparison of the Optimized L4 Monomer With Previously Characterized Peptides for Cargo Uptake

Having identified the L4 mutant from our screening, we first evaluated its dose–response profile to establish the optimal working concentration. Treating cells with varying concentrations of the new L4 monomer (peptide 10) alongside a fixed amount of NA‐650 resulted in a clear dose‐dependent increase in cargo uptake, reaching its maximum efficiency at 500 nM (Figure 6B).

**FIGURE 6:**
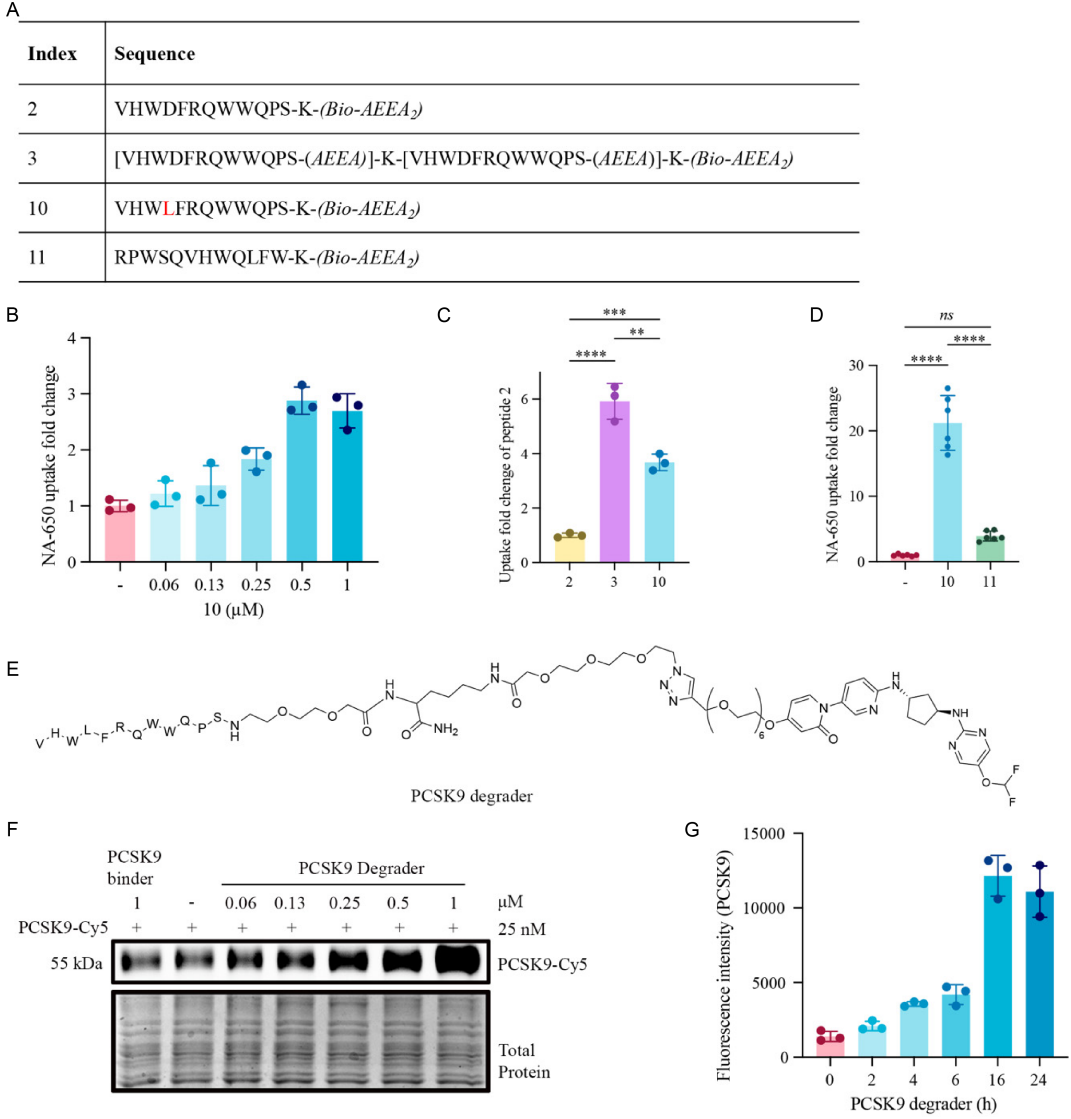
Evaluation of the optimized L4 monomer and its application in targeted PCSK9 internalization. (A) Amino acid sequences of the synthesized biotinylated peptides. The specific single leucine substitution in the optimized monomer (peptide 10) is highlighted in red, and peptide 11 serves as a randomized sequence control. (B) Dose‐dependent cellular uptake of NA‐650 (25 nM) cargo mediated by peptide 10. Data are presented as mean ± SD (*n* = 3 biological independent samples). (C) Comparison of recruitment efficiency of NA‐650 (25 nM) among the wild‐type monomer (peptide 2), the bivalent dimer (peptide 3), and the optimized monomer (peptide 10) at 500 nM. Data are presented as mean ± SD. The statistical significance was assessed using ordinary one‐way ANOVA test, **p < 0.01, ***p < 0.001, and ****p < 0.0001 (*n* = 3 biological independent samples). (D) Comparison of NA‐650 (125 nM) cellular uptake mediated by peptide 10 and the scrambled control (peptide 11) at 500 nM. Data are presented as mean ± SD. The statistical significance was assessed using ordinary one‐way ANOVA test, ****p < 0.0001, and ns: not significant (*n* = 3 biological independent samples). (E) Chemical structure of the bifunctional molecule targeting PCSK9, comprising the optimized L4 recruitment peptide conjugated to the specific PCSK9 inhibitor AZD0780 via a chemical linker. (F) Dose‐dependent cellular internalization of Cy5‐labeled PCSK9 (25 nM) driven by the bifunctional molecule targeting PCSK9 for 4 h treatment in Huh7 cells. (*n* = 3 biological independent samples). (G) Time‐course evaluation of PCSK9 (25 nM) internalization mediated by the bifunctional molecule (500 nM), demonstrating a continuous, time‐dependent increase in cellular fluorescence intensity over 24 h. Data are presented as mean ± SD (*n* = 3 biological independent samples).

Utilizing this optimal 500 nM concentration, we next compared its recruitment efficiency against our established biotinylated peptides (Figure 6C). Peptide 10 induced a substantial increase in cargo uptake, achieving a three to fourfold enhancement compared to the wild‐type monomer (peptide 2). Although its internalization efficiency remained lower than that of the bivalent peptide 3, this single amino acid substitution significantly bridged the performance gap, offering a highly advantageous balance between molecular simplicity and functional potency.

Furthermore, to confirm sequence specificity, we synthesized a control peptide (peptide 11) that retains the identical amino acid composition but features a randomized sequence. While peptide 10 facilitated robust cargo uptake at 500 nM, peptide 11 induced only marginal internalization (Figure 6D), confirming that the dramatic enhancement in efficacy is driven by the specific sequence rather than general physicochemical properties. However, to explicitly verify whether Peitide 10‐mediated uptake remained dependent on IGF2R, we compared the internalization of peptide 10 in wild‐type Huh7 cells versus IGF2R‐KD Huh7 cells. In contrast to the parental peptide, whose uptake was reduced following IGF2R KD, peptide 10‐mediated cargo uptake was not detectably affected under the same conditions. However, because IGF2R expression was only partially reduced in the KD cells, the current data do not exclude the possibility that the enhanced activity of peptide 10 enables efficient utilization of the remaining IGF2R pool. Thus, these results indicate that peptide 10‐mediated uptake is less sensitive to partial IGF2R depletion, while the precise receptor dependance of this variant remains to be established.

Together, these experiments demonstrate that peptide 10 mediates highly efficient and sequence‐specific cargo internalization. Its enhanced uptake activity and structurally simple monomeric architecture establish it as a promising building block for future application in TPD systems.

### 2.7 Targeted Internalization of PCSK9 Using an L4‐Based Bifunctional Molecule

Having validated the efficiency of the L4 monomer (peptide 10) using a model cargo, we next evaluated its applicability against a therapeutically relevant target. We selected Proprotein Convertase Subtilisin/Kexin type 9 (PCSK9), a well‐established target in cardiovascular disease [25]. PCSK9 promotes the lysosomal degradation of LDLR to inhibit the plasma clearance of low‍‐‍‍density lipoprotein cholesterol (LDL‐C). Elevated serum LDL‐C levels are the major risk factor for CVD, one of the leading causes of death worldwide [26]. To achieve this, we synthesized a bifunctional molecule by conjugating the optimized L4 recruiter to AZD0780, a potent and clinically relevant PCSK9‐binding ligand, to facilitate the targeted clearance of this cholesterol‐regulating protein. (Figure 6E) [27, 28].

To assess the efficacy of this L4‐based construct, we monitored the cellular uptake of PCSK9. Treating cells with the bifunctional molecule resulted in dose‐dependent internalization of PCSK9 (Figure 6F). Time‐course assays further confirmed a time‐dependent accumulation of intracellular PCSK9 following treatment (Figure 6G). These data demonstrate that the L4 recruiter can be successfully adapted to drive the uptake of an endogenous disease‐relevant protein.

## 3 Conclusion

In this study, we first evaluated a previously reported IGF2R‐binding peptide (431). We validated the robust cargo internalization of both its monomeric and dimeric forms. Beyond cargo internalization, we also examined the postinternalization fate of recruited protein. Following washout of the bifunctional molecule, the intracellular cargo signal (NA‐650) progressively decreased over time, indicating subsequent intracellular clearance rather than stable accumulation. Together with our lysosomal colocalization studies using NeutrAvidin as a model cargo, these findings support the ability of the optimized peptide scaffold to promote cargo uptake and lysosomal trafficking. Furthermore, we explicitly confirmed the IGF2R‐dependent internalization induced by the bivalent dimer, establishing the wild‐type peptide scaffold as an effective and functional baseline for IGF2R‐targeted LYTACs.

Building upon this established mechanism, we then optimize the monomeric sequence to simplify the recruiter architecture. Through mutational screening, we identified the L4 variant (peptide 10), which features a single leucine substitution at the fourth position. This optimized monomer exhibited a substantial increase in endocytic activity compared to the wild‐type scaffold. While its cellular uptake was slightly lower than that of the bivalent dimer, it delivered robust and highly efficient internalization. Finally, to extend our findings to a therapeutically relevant target, we constructed a bifunctional molecule by conjugating the L4 recruiter with a PCSK9 binder, which successfully induced the dose‐ and time‐dependent internalization of PCSK9.

A scrambled control peptide with an identical amino acid composition confirmed that the enhanced uptake mediated by L4 is sequence‐specific. IGF2R KD studies further showed that L4‐mediated internalization was less sensitive to partial IGF2R depletion than uptake mediated by the parental peptide. However, because substantial residual IGF2R remained after KD, these data cannot distinguish between efficient utilization of the remaining IGF2R pool and a possible contribution from additional uptake pathways. More stringent approaches, including complete IGF2R knockout models and unbiased genetic screening, will therefore be valuable for defining the receptor dependance and endocytic mechanisms underlying the enhanced activity of L4.

Finally, to extend this platform toward a therapeutically relevant target, we conjugated the L4 recruiter to a PCSK9 binder. The resulting bifunctional molecule induced dose‐ and time‐dependent internalization of PCSK9, demonstrating that the opti‍mized recruiter can be incorporated into a target‐specific bifunctional architecture. Future studies will extend the L4 platform to additional therapeutically relevant extracellular and membrane‐associated proteins. Further optimization of the target‐binding moiety, linker architecture, recruiter valency, and overall molecular configuration may enhance internalization efficiency and broaden the applicability of this peptide‐based LYTAC platform.

## Funding

This work was supported by the National Institutes of Health (Grant NIGMS R35GM148266).

## Conflicts of Interest

W.T. is the cofounder and shareholder of Chimergen Therapeutics, Inc., NovaCure Biosciences Holding Inc., MolTenon Biosciences Inc., and GlycoBridge Biosciences Inc.

## Supporting information

Supplementary Material

## Data Availability

All data are in the manuscript and supporting information.
