# Supplementary material for: Development of Highly Efficient Lysosome‐Targeting Peptide Ligands for the Internalization of Extracellular Proteins
Source: Chembiochem. 2026 Oct 28;27(21):e70587. doi: 10.1002/cbic.70587 (PMC13717051; doi:10.1002/cbic.70587)
Supplement: Supplementary file 1 [file CBIC-27-e70587-s001.pdf]

Development of Highly Efficient Lysosome-Targeting Peptide Ligands for the Internalization of Extracellular Proteins

Yuan Zhao †, Yaxian Liao †, Xuankun Chen†, Deqin Cai, Regina Stasser de Gonzalez, Hua Tang, Florence M. Brunel, Nick Cox, Joseph Stock, Guangsen Fu, Alexander N. Zaykov,* Weiping Tang*

[a] Y. Zhao, X. Chen, Dr. D. Cai, R. Stasser de Gonzalez, Dr. H Tang, Prof. Dr. W. Tang
Lachman Institute of Pharmaceutical Development, School of Pharmacy, University of Wisconsin-Madison, Madison, WI, 53705, USA; E-mail: weiping.tang@wisc.edu

[b] Y. Liao, Prof. Dr. W. Tang
Department of Chemistry, University of Wisconsin-Madison, Madison, WI, 53706, USA

[c] Dr. F. M. Brunel, Dr. N. Cox, Dr. J. Stock, Dr. G. Fu, Dr. A. N. Zaykov
Chemical Biology, Novo Nordisk US R&D, 33 Hayden Ave., Lexington, MA 02421, USA; E-mail: azaykov@penguin.bio

Table Contents:

Figure S1. Comparison of NA-650 internalization induced by biotinylated peptides.

Figure S2. Structure-activity relationship (SAR) analysis of dimeric IGF2R recruiters in U87 cells.

Figure S3. Characterization of Peptide 2-mediated cargo internalization.

Figure S4. Characterization of biotinylated peptides.

Figure S5. HRMS of PCSK9 degrader**.**

Experimental Section

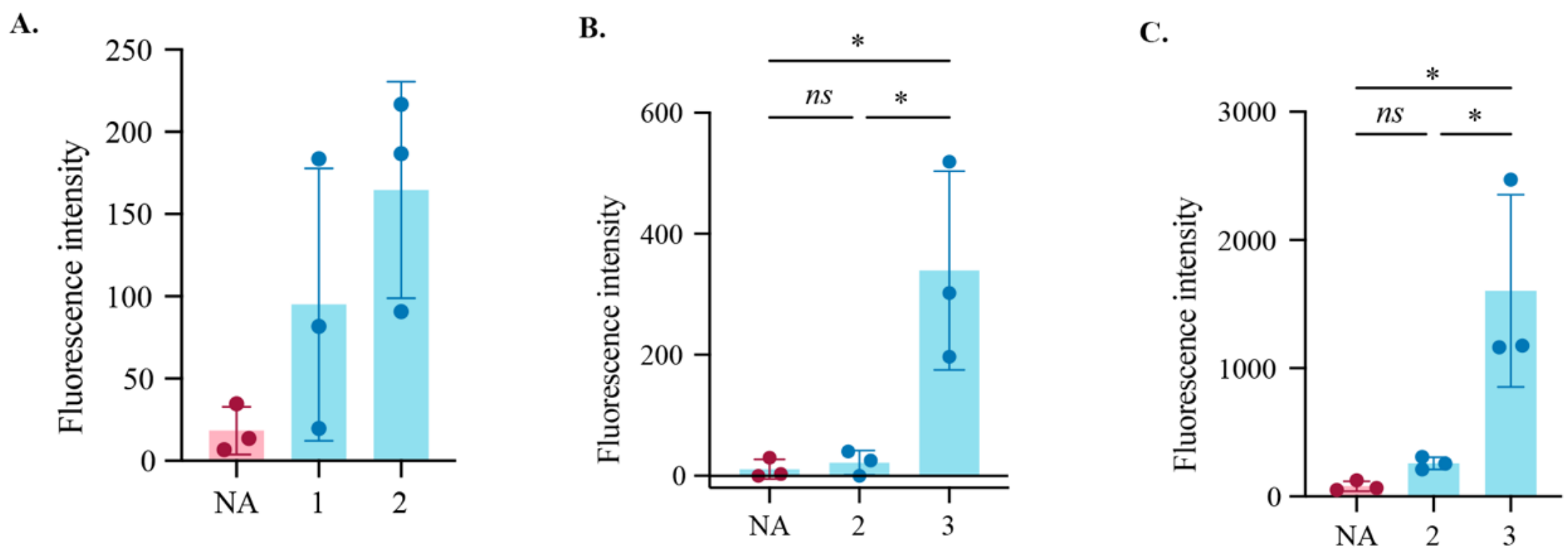

Figure S1. Comparison of NA-650 internalization induced by biotinylated peptides. **(A)** Comparison of cellular uptake of the model cargo (NA-650, 125 nM) driven by Peptides 1 (500 nM) and 2 (500 nM) in U87 cell line. Data are presented as mean ± SD. (n = 3 biological independent samples). **(B, C)** Comparison of recruitment efficiency between the monomeric Peptide 2 (500 nM) and the dimeric Peptide 3 (500 nM) at varied cargo concentrations (25 nM (**B**) and 125 nM (**C**)) in U87 cells. Data are presented as mean ± SD. The statistical significance was assessed using ordinary one-way ANOVA test, **p* < 0.05, ns: not significant (n = 3 biological independent samples).

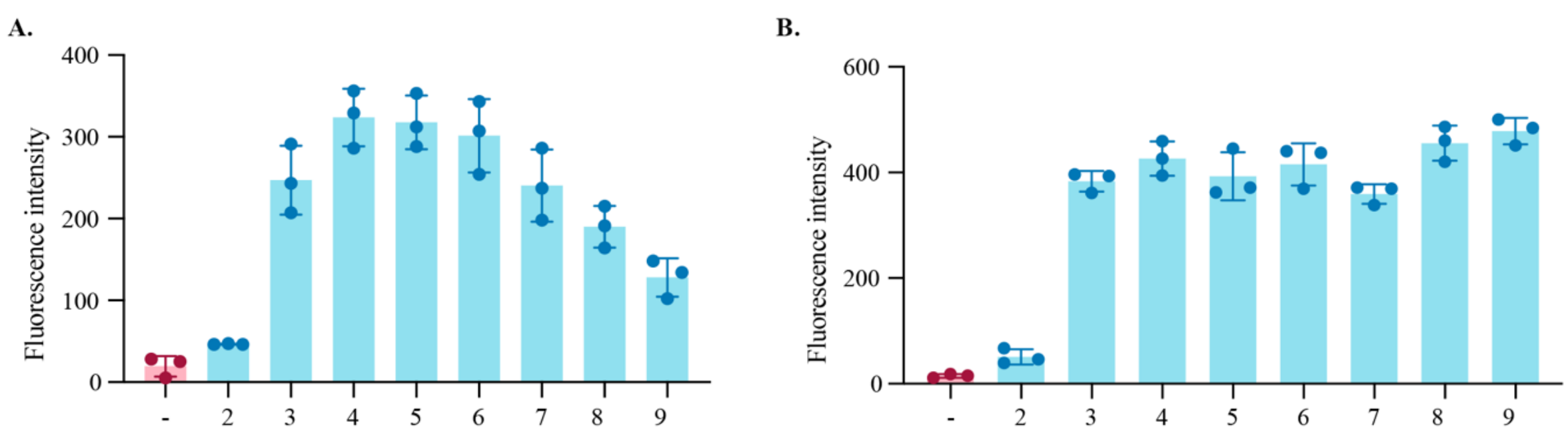

Figure S2. Structure-activity relationship (SAR) analysis of dimeric IGF2R recruiters in U87 cells. (**A, B**) Fluorescence quantification via microplate reader comparing cellular uptake of model cargo (NA-650, 25nM) driven by dimeric variants 4-9 in U87 cells at two concentrations: 100 nM (**A**) and 500 nM (**B**). Peptide 2 (WT monomer) and Peptide 3 (WT dimer) serve as benchmarking controls. Data are presented as mean ± SD (n = 3 biological independent samples).

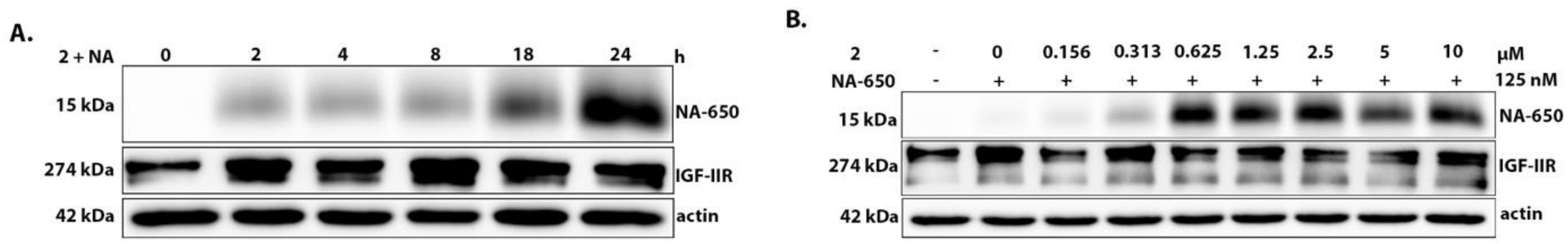

Figure S3. Characterization of Peptide 2-mediated cargo internalization. **(A)** Time-course of NA-650 (125 nM) cellular uptake (0–24 h) driven by Peptide 2 (500 nM) in HepG2 cells. (n = 3 biological independent samples).**(B)** Concentration-dependent internalization of NA-650 in HepG2 cells treated with varied concentrations of Peptide 2 (0–10 μM) and fixed NA-650 (125 nM). (n = 3 biological independent samples).

Index	Calculated Mass	Found Mass
1	([M+H]3+) 730.01	730.05
2	([M+H]2+) 1158.56	1158.4
3	([M+H]4+) 1097.29	1097.28
4	([M+H]3+) 1308.98	1308.97
5	([M+H]3+) 1367.00	1367.12
6	([M+H]4+) 1170.58	1170.71
7	([M+H]7+) 751.37	752.40
8	([M+H]4+) 1153.85	1153.93
9	([M+H]5+) 945.68	945.68
10	([M+H]3+) 917.13	917.04
11	([M+H]3+) 917.13	917.04

Figure S4. Characterization of biotinylated peptides.

HRMS of PCSK9 Degrader

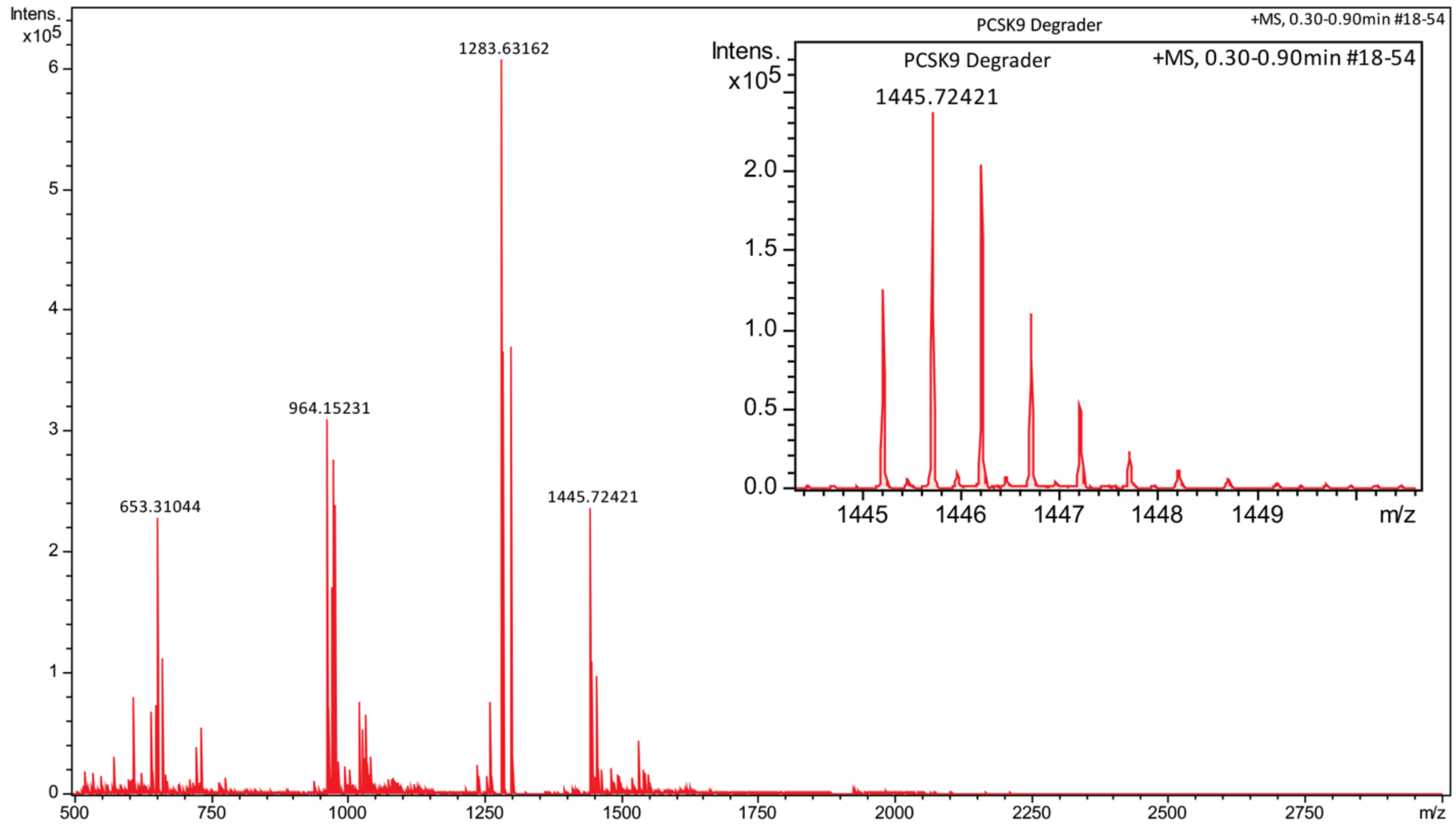

Figure S5. HRMS of PCSK9 degrader**.**

Experimental Section

Cell culture

U87 (RRID CVCL_0022) cells were cultured in high-glucose Dulbecco's Modified Eagle Medium (DMEM) supplemented with 10% fetal bovine serum (FBS) and 1% penicillin-streptomycin. HepG2 (RRID CVCL_0027) and Huh7 (RRID CVCL_0336) cells were maintained in low-glucose DMEM supplemented with 10% FBS, 1% penicillin-streptomycin, 1% non-essential amino acids (NEAA), 1% sodium pyruvate, and 1% L-glutamine. All cell lines were incubated at 37 °C in a humidified atmosphere containing 5% CO2.

Peptides

All biotinylated peptides used in this manuscript are provided by Novo Nordisk.

Quantification of NA-650 Cellular Uptake via Microplate Reader

Cells were seeded into 96-well plates and cultured overnight to allow for adherence. The following day, the cells were co-incubated with the indicated biotinylated peptides and NA-650 for the specified time periods. Following treatment, the cells were washed three times with phosphate-buffered saline (PBS) to remove excess extracellular reagents. Cellular fluorescence intensity was subsequently measured using

a Synergy H1 microplate reader (excitation: 650 nm, emission: 680 nm) to determine the uptake efficiency.

In-Gel Fluorescence Assay for NA-650 Cellular Uptake

Cells were seeded into 48-well plates and cultured overnight to allow for adherence. The following day, the cells were co-incubated with the indicated biotinylated peptides and NA-650 for the specified time periods. Following treatment, the cells were washed three times with phosphate-buffered saline (PBS) and immediately lysed in 1× RIPA buffer (25 mM Tris, pH 7.0–8.0, 150 mM NaCl, 0.1% (w/v) SDS, 0.5% sodium deoxycholate, 1% (v/v) Triton X-100) supplemented with protease inhibitors. The lysates were incubated on ice for 15 min, harvested into 1.5 mL microcentrifuge tubes, and cleared by centrifugation at 16,000 × *g* for 15 min at 4 °C. The supernatants were collected into new tubes, and protein concentrations were determined using a BCA protein assay. Based on the BCA results, all samples were normalized to equal total protein amounts and volumes using ddH_2O. Finally, 4× Laemmli loading buffer was added, and the samples were denatured by boiling at 99 °C for 5 min before being resolved by 7.5% or 12.5% SDS-polyacrylamide gel electrophoresis (SDS-PAGE) for subsequent fluorescence visualization.

Quantification of NA-650 Depletion from Culture Media

Huh7 cells were seeded into 48-well plates and cultured overnight to allow for adherence. The following day, the growth medium was aspirated, and the cells were treated with 100 µL of serum-free medium containing NA-650 and the indicated degraders. After the designated incubation period, a 30 µL aliquot of the conditioned serum-free medium was harvested from each well and subjected to in-gel fluorescence analysis to evaluate the extracellular depletion of NA-650.

Confocal Imaging for NA-650 Colocalization

Huh7 cells were plated in 8-well chamber slides and incubated overnight. The cells were then treated with peptide 3 and NA-650 for 24 h. The cells were sequentially stained with 1 µM LysoTracker (30 min) and Hoechst (10 min). After washing three times with PBS to remove excess dyes, the slides were imaged on a Leica SP8 3X STED super-resolution microscope using a 60× objective. All acquired images were analyzed using ImageJ software.

CRISPR/Cas9-Mediated Disruption of IGF-IIR in Huh7 Cells

The IGF-IIR disrupted Huh7 single-cell clones (IGF-IIR-/+) were established as previously described[23] . Briefly, Huh7 cells were seeded into 24-well plates and cultured overnight. The cells were then transfected with an IGF-IIR-targeting sgRNA plasmid (Horizon Discovery) using FuGENE reagent in Opti-MEM medium. After 48 h, the transfection medium was replaced with standard growth medium supplemented with 2 µg/mL puromycin. The selection medium was refreshed every other day for two weeks. Surviving cells were trypsinized and sparsely seeded into 100-mm dishes in the presence of 1 µg/mL puromycin to isolate single-cell clones. Individual clones were subsequently expanded in 6-well plates and transferred to 100-mm dishes upon reaching 80% confluence, where they were continuously maintained in medium containing 1 µg/mL puromycin.

Quantification of Cell Surface IGF-IIR via Flow Cytometry

The cell surface expression of IGF-IIR was evaluated via flow cytometry. Briefly, cultured cells were detached using trypsin-EDTA, washed three times with PBS, and incubated with an anti-IGF-IIR primary antibody (ab124767, Abcam) for 30 min on ice. Following three additional washes with PBS, the cells were incubated with an Alexa Fluor 647-conjugated anti-rabbit secondary antibody (A-21244, Thermo

Fisher Scientific). After a final three washes with PBS, the cells were resuspended in 500 µL of PBS containing 1% BSA and DAPI for dead cell exclusion. Data acquisition was performed on a Thermo Fisher Attune NxT flow cytometer, and the data were analyzed using FlowJo software.

PCSK9 degrader synthesis

The synthesis of this conjugate is achieved by coupling standard Fmoc-based solid-phase peptide synthesis (SPPS) and one step click chemistry[20] . First, the D4 peptide sequence (VHWLFRQWWQPS) is assembled on a Rink Amide resin, followed by the coupling of a PEG-spacer and an azide-functionalized building block; the resulting azido-peptide is then cleaved and deprotected using a TFA/H_2O/TIPS = 95/2.5/2.5 cocktail. Then, the azide modified peptide was further coupled with PCSK9 binder with an alkyne-terminated linker in the presence of $CuSO_4$ (2.0 e.q.), sodium ascorbate (2.0 e.q.) and tris((1-benzyl-4-triazolyl)methyl)amine (TBTA, 2.0 e.q.). The reaction mixture was purified by preparative HPLC C18 reverse column eluting with acetonitrile/water (0.1% TFA) and characterized by ESI-MS. HRMS (ESI) for $C_{138}H_{191}F_2N_{35}O_{32}$ [M+H]2+ 1445.2254 (Calc.), found 1445.2224.

Quantification of PCSK9-Cy5 Cellular Uptake

Huh7 cells were seeded into 48-well or 96-well plates and cultured overnight to ensure complete adherence. The following day, the culture medium was replaced with fresh medium containing Cy5-conjugated PCSK9 (PCSK9-Cy5) and the indicated degraders for specified incubation periods. After treatment, the cells were washed three times with PBS to thoroughly remove any non-internalized or membrane-bound PCSK9-Cy5. For quantitative assessment using a microplate reader, the fluorescence intensity of the internalized PCSK9-Cy5 was measured directly or after cell lysis (Ex/Em = 649/670 nm). Alternatively, for in-gel fluorescence analysis, cells were harvested and lysed as previously described. The resulting protein samples were normalized and resolved by SDS-PAGE, followed by fluorescence visualization using a gel imaging system to evaluate the intracellular accumulation of PCSK9.

Statistical Analysis

Quantitative results are expressed as the mean ± standard deviation (SD). All statistical evaluations were conducted utilizing GraphPad Prism software. Differences between experimental conditions were assessed using an unpaired, two-tailed t-test (for two groups) or an ordinary one-way analysis of variance (ANOVA) followed by Dunnett’s post hoc test (for multiple groups). A threshold of $p < 0.05$ was defined as statistically significant.

References:

See manuscript for references 20 and 23.
